# In silico simulation of glycosylation and related pathways

**DOI:** 10.1007/s00216-024-05331-8

**Published:** 2024-05-15

**Authors:** Yukie Akune-Taylor, Akane Kon, Kiyoko F. Aoki-Kinoshita

**Affiliations:** 1 Glycan and Life Systems Integration Center, Soka University, Tokyo, Japan; 2 Graduate School of Science and Engineering, Soka University, Tokyo, Japan; 3 iGCORE, Nagoya University, Nagoya, Japan

**Keywords:** Glycosylation, Bioinformatics, Systems biology, Pathways, Prediction, Software

## Abstract

Glycans participate in a vast number of recognition systems in diverse organisms in health and in disease. However, glycans cannot be sequenced because there is no sequencer technology that can fully characterize them. There is no “template” for replicating glycans as there are for amino acids and nucleic acids. Instead, glycans are synthesized by a complicated orchestration of multitudes of glycosyltransferases and glycosidases. Thus glycans can vary greatly in structure, but they are not genetically reproducible and are usually isolated in minute amounts. To characterize (sequence) the glycome (defined as the glycans in a particular organism, tissue, cell, or protein), glycosylation pathway prediction using in silico methods based on glycogene expression data, and glycosylation simulations have been attempted. Since many of the mammalian glycogenes have been identified and cloned, it has become possible to predict the glycan biosynthesis pathway in these systems. By then incorporating systems biology and bioprocessing technologies to these pathway models, given the right enzymatic parameters including enzyme and substrate concentrations and kinetic reaction parameters, it is possible to predict the potentially synthesized glycans in the pathway. This review presents information on the data resources that are currently available to enable in silico simulations of glycosylation and related pathways. Then some of the software tools that have been developed in the past to simulate and analyze glycosylation pathways will be described, followed by a summary and vision for the future developments and research directions in this area.

**Graphical Abstract:**

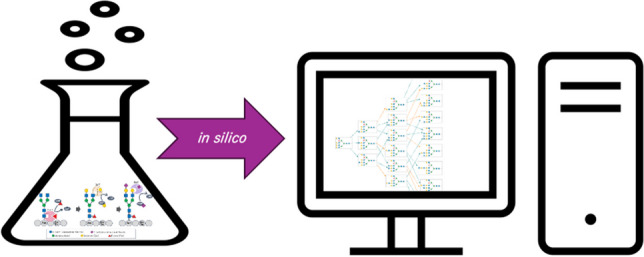

## Introduction

Glycans, also called polysaccharides or carbohydrates, are chains of variously linked monosaccharides biosynthesized by glycosyltransferases. They can occur as free oligosaccharides or as parts of glycoproteins and glycolipids. They participate in a vast number of recognition systems in diverse organisms in health (development, cell differentiation, inflammation, signaling, and immunomodulation) and in disease (infectious and non-infectious including neoplasia) [1]. They are known to be involved in virus infection, including influenza [2] and even SARS-CoV-2 [3], as they cover the spike protein of this latter virus. The reason why they are so involved in many biological processes is that they are found on the cell surface of practically every cell in the body. They are attached to proteins and lipids on the cell surface, and sometimes, they are secreted outside of the cell. They are also involved in the extracellular matrix as proteoglycans; well-known proteoglycans are heparan sulfate, chondroitin sulfate, and keratan sulfate [4].

**Fig. 1:**
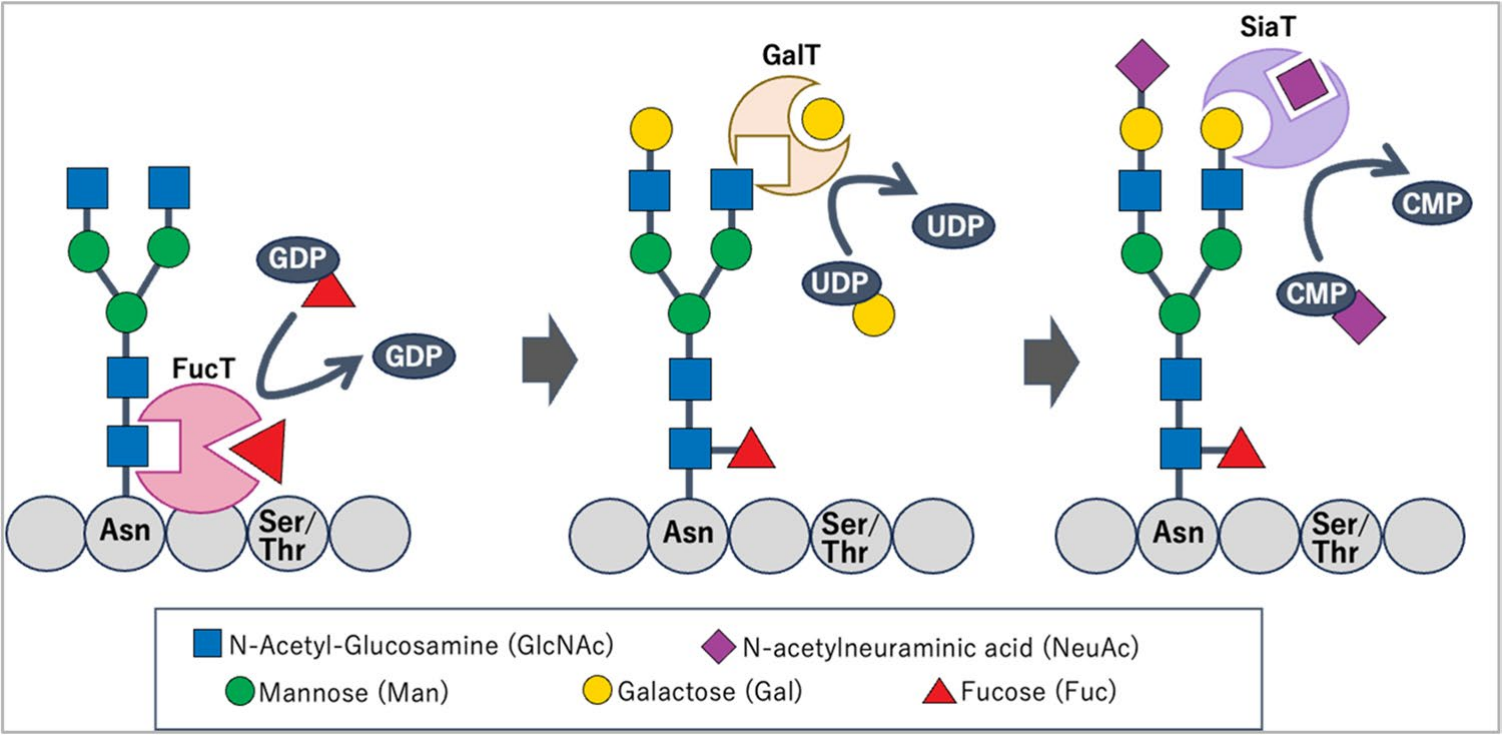
An example scheme of a biosynthetic pathway of a glycan being processed in the Golgi apparatus. Various glycosidases and glycosyltransferases (here, FucT, GalT, and SiaT, which transfer a fucose residue, a galactose residue, and a sialic acid residue, respectively, from their corresponding nucleotide-sugar) remove or add monosaccharides to synthesize a glycan

Glycans cannot be sequenced as proteins or DNA due to the absence of a fully characterizing sequencer technology and a replicating template. Instead, glycans are synthesized by a complicated orchestration of hundreds of glycosyltransferases, glycosidases, and other enzymes such as epimerases and sulfotransferases (often collectively termed “glycogenes”) localized widely from the endoplasmic reticulum, through the Golgi apparatus, and out to the trans-Golgi network. For example, an *N*-glycan is first extended in the cytoplasmic side of the ER to form Man9 with glucose-caps. It is then flipped to the lumenal side of the ER and transferred to an asparagine residue of a protein. The glucose-caps and mannoses are then trimmed by glucosidase I and II and mannosidase I (*N*-glycan precursor). When the proteins are transferred into the Golgi apparatus, the *N*-glycan precursor is further processed and modified by glycosyltransferases such as FucT, GalT, and SiaT (Fig. 1). The CAZy database encompasses more than 300 families of glycosidases and glycosyltransferases. Each of these enzyme families represents distinct modules with different substrate specificities and reaction conditions. Thus, glycans can vary greatly in structure. For example, bacterial glycans, such as those found on *N*-glycoproteins and lipopolysaccharides, are mainly found on the cell wall. They are involved in host-interactions and pathogenesis. Plant glycans, such as cellulose and pectin, play a key role in cell wall stabilization in terms of its strength and resistance to various environmental stresses. The differences in the functions and structures of glycans in each biological field are due to evolutionary and ecological factors, which have shaped the strategies of each organism to adapt to different environments. Therefore, it is crucial, when using software tools, to grasp these distinctions for a precise assessment of tool applicability.

To characterize (sequence) glycans, no next-generation sequencer exists, and so current technologies include mass spectrometry, liquid chromatography, and nuclear magnetic resonance. All these technologies require highly technical skills and sufficient amounts of samples to analyze. Therefore, other means of characterizing the glycome (defined as the glycans in a particular organism, tissue, cell, or protein) have been developed. This includes calculation algorithms to measure the physicochemical similarities of glycan structures, glycosylation pathway prediction based on glycogene expression data, and glycosylation simulation. In particular, many of the mammalian glycogenes have been identified, cloned, and their activities identified, so it has become possible to predict the glycan biosynthesis pathways in silico using these data. In the last decade, these in silico models have been rapidly improved by incorporating multiomics data (genomics, transcriptomics, and proteomics), systems biology, and bioprocessing technologies to these pathway models. Furthermore, the addition of appropriate enzymatic parameters including enzyme and substrate concentrations and kinetic reaction parameters enable the prediction of the potentially biosynthesized glycans that are involved in specific pathways such those involved in health and disease.

This article will present information on currently available data resources used for in silico simulations of glycosylation and related pathways. These include Web tools and programming libraries for pathway modeling that have been made available for users to utilize and or expand them for their own experimental data. There are also databases that have accumulated various information relevant to glycogenes. Then some of the software tools that have been developed to take a step further and perform simulations and analyze glycosylation pathways will be presented, followed by a summary and vision for the future developments and research directions in this area.

## Data resources

There are several data resources that provide information on glycogenes. CAZy (Carbohydrate-Active enZymes) [5] is one of the oldest carbohydrate-related databases still running, having started circa 1998. It organizes information on carbohydrate enzymes involved in glycosylation, metabolism, and transportation of glycans into six classes, each of which are subdivided into families, the numbers of which continue to grow, but at the time of this writing number as follows:

1. Glycoside hydrolases (GH), 185 families
2. Glycosyltransferases (GT), 116 families
3. Polysaccharide lyases (PL), 42 families
4. Carbohydrate esterases (CE), 20 families
5. Auxiliary activities (AA), 16 families
6. Carbohydrate-binding modules (CBM), 98 families

CAZy enzymes (often called CAZymes) are classified based on their amino acid sequence similarities as there are correlations between sequence and protein folding similarities. Many of the families are then classified based on the three-dimensional patterns of the protein structures. CAZy provides mutual links with KEGG, RCSB PDB, Expasy, and other databases. Many of the CAZymes are automatically populated based on the sequences registered into NCBI GenBank.

The GlycoGene Database (GGDB) [6] was originally developed under the Japanese government-funded Glycogene Project (GG Project) in 2001. Over 180 genes of human glycosyltransferases and sulfotransferases were cloned and recorded into GGDB. Each entry page is manually curated and includes Gene ID, DNA sequences, tissue distribution of gene expression, substrate specificity, homologous genes, and external links to other databases such as GenBank and CAZy. The latest data in GGDB are now available in the ACGG-DB database (https://acgg.asia/db/), which are integrated into the GlyCosmos Glycoscience Portal [7].

KEGG is well-known as a Web resource for biological systems data including genomic, chemical, and health and disease information. It is a major provider of manually curated pathways, as well as databases for genes and genomes. It also includes a GLYCAN resource [8], which contains glycan structures that participate in the glycan-related pathways stored in KEGG, as well as glycogene information, often annotated with E.C. numbers. Most recently, disease-related information related to glycogenes have been incorporated into KEGG.

In the field of bioprocessing, Chinese Hamster Ovary (CHO) cells provide a standard platform for production of protein therapeutics because of their human-like glycosylation. Thus, CHOGlycoNET [9] was developed as a comprehensive network encompassing glycosylation reactions that account for all experimentally observed glycans found in recombinant proteins and both intracellular, membrane, and secreted host cell proteins within two major CHO cell lineages, namely CHO–S and CHO–K1. This is the largest dataset of CHO cell glyco-profiles comprising 200 datasets sourced from seven different laboratories; it serves as the basis for uncovering potential latent reactions that could become active under a variety of genetic glycoengineering and metabolic perturbation scenarios, for a range of recombinant glycoproteins and CHO cell host cell proteins.

## Software and tools

Many software tools have been developed in the past, even when there were few datasets readily available for analyzing glycogenes. Here, we describe software tools for glycan biosynthesis analysis and for in silico simulation for the prediction of glycomes.

### Glycan biosynthesis prediction

Glycologue (https://glycologue.org/) is a Web portal for glycosylation prediction tools for *N*- and *O*-glycans, human-milk oligosaccharides and gangliosides [10–12]. Users can simulate the glycosylation pathway by choosing a starting glycan structure and selecting glycosyltransferases from a predefined list. This list has been manually curated and includes the reaction pattern representing the substrate specificity of the given glycogene. The model then calculates the glycosylation pathway using the selected enzymes. It is also possible to calculate a minimal set of glycosyltransferases to biosynthesize the starting glycan structure. Using this model, the authors were able to predict a highly heterogeneous set of structures when all O-glycan-related enzymes (25 glycosyltransferase and sulfotransferase enzymes) were allowed to act, including many clinically important epitopes such as Sialyl-Lewis X. Moreover, in silico knockout experiments were performed, and they were able to achieve 98% coverage of glycans predicted for specific knockout cell lines.

GlycoVis is a visualization tool designed to illustrate the distribution of N-glycans within a reaction network, along with the potential pathways for reactions associated with each glycan [13]. The enzyme substrate specificities have been structured into a matrix of relationships. Upon inputting glycan distribution data, the program generates a pathway map that represents various glycans using distinct colors to indicate their relative abundance levels. Additionally, it identifies and traces all feasible reaction routes leading to each glycan on the map. To demonstrate GlycoVis’s utility, it was applied to illustrate the glycoform distribution in Chinese Hamster Ovary (CHO) cell-derived tissue plasminogen activator (TPA), as well as human and mouse IgG.

**Fig. 2:**
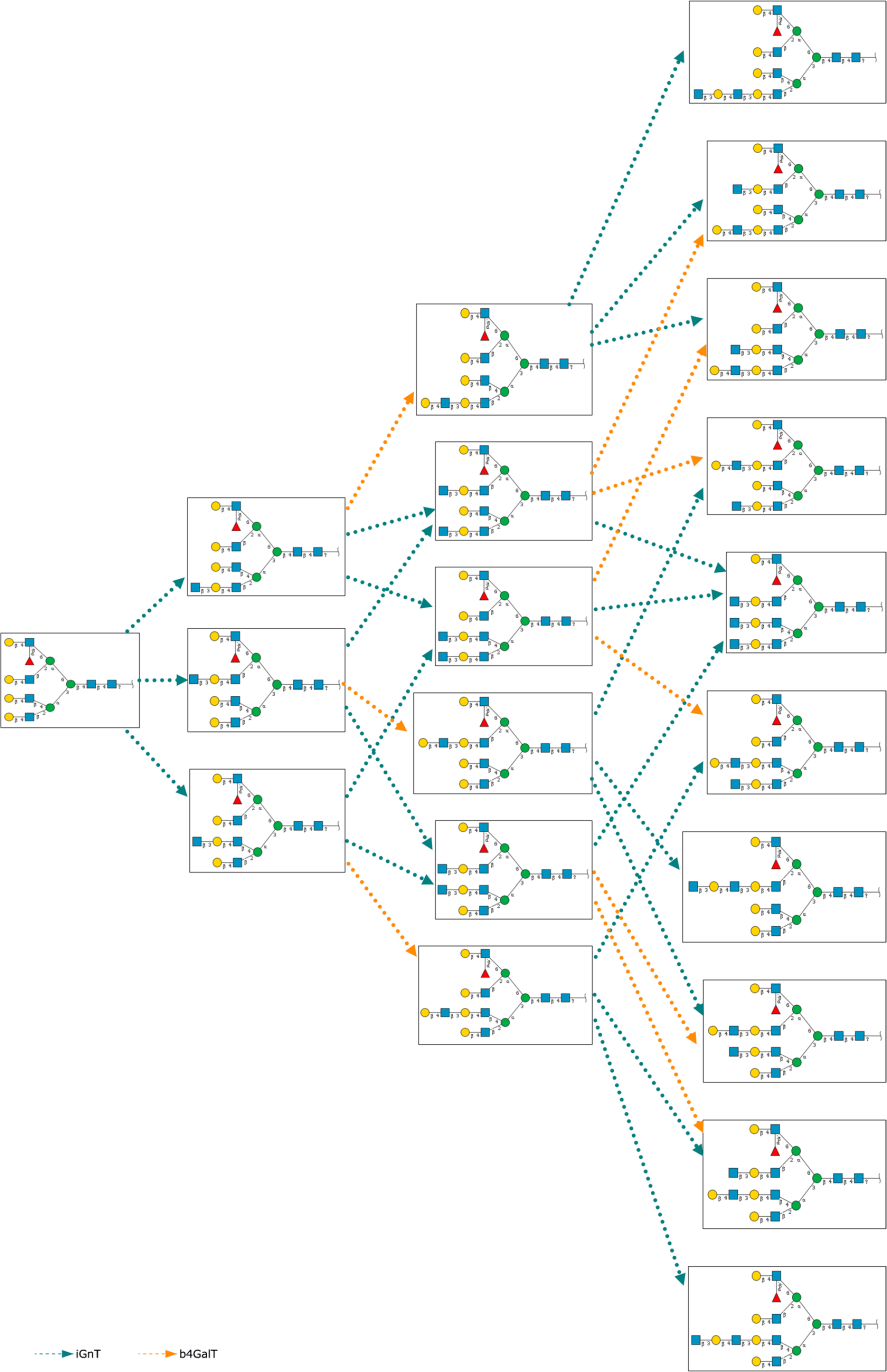
An example of the resulting page from running the Glycan Pathway Predictor (GPP) tool in RINGS. All the potentially biosynthesized glycans using just two genes, iGnT and b4GalT, from a single tetra-antennary N-glycan structure are shown, indicating the complexity of glycan biosynthesis

Glycan Pathway Predictor (GPP) [14] is a Web tool to predict* N*-glycosylation pathways, given a starting glycan and a selected list of glycogenes; the computation is based on a mathematical model proposed earlier [15, 16]. It is available on the RINGS (https://rings.glycoinfo.org) resource [17]. A total of 19 glycosyltransferases are available by default, and users can also limit the size of the predicted pathway by specifying the maximum mass of glycans to predict. For example, Fig. 2 shows a snapshot of the results of predicting the biosynthetic pathway using just two genes iGnT and b4GalT starting from a single tetra-antennary *N*-glycan structure. The figure shows 19 glycans and 30 reactions that could potentially take place. This model does not take cellular localization into consideration, but constraints on the substrate specificity of each gene can be made to emulate such information.

UniCorn [18] is a database developed from the results of utilizing GPP to predict the glycosylation pathway of *N*-glycans (> 15 monosaccharide residues) using 45 human glycosyltransferases. Enzyme specificities were extracted from KEGG, CFG, CAZy, GGDB, and BRENDA [19]. As a result, more than 1.1 million theoretical structures and 4.7 million synthetic reactions were generated and stored in UniCorn, which was made available in UniCarbKB [20]. Similarly to Fig. 2, a tremendous amount of glycans can be potentially biosynthesized from a handful of glycans, but in reality, based on information deposited in glycan structure databases, only a few thousand have actually been identified. Therefore, more research into the cellular localization and structure of the Golgi apparatus, where most of the glycogenes reside, need to be made to better model these pathways.

VirtualGlycome (https://virtualglycome.org) is a Web portal developed by Neelamegham et al. to provide software tools and experimental resources for glycan structure analysis. Currently, five software tools are provided: DrawGlycan-SNFG [21], GNAT [22], and GlycoPAT [23], among others. GNAT, in particular, is a free open-source MATLAB toolbox for predicting glycosylation networks. It provides various functionality, including network prediction, similar to GPP, to reconstruct glycosylation networks given a set of reactions and/or products and a list of enzymes; prediction of networks from mass spectrometry data; and dynamic and steady-state simulations of reaction networks. While this software has not been updated in a while, it is still available for download, and the author is reachable for questions.

GlycoMME (Glycosylation Markov Model Evaluator) [24] is a toolkit for analyzing the effect of glycoengineering on the theoretical *N*-glycosylation biosynthesis. They facilitated *N*-glycosylation as a Markov model to quantify the specificity of isozymes and the interactions of glycosyltransferases, which helps users to predict the *N*-glycosylation process.

GlycoCompare [25] is a computational approach for the rapid and scalable analysis of comparing multiple glyco-profiles. It calculates the glycan intermediates, which are used as interpretable functional units, to address the hidden interdependencies between glycomics samples. The authors demonstrated the GlycoCompare method using recombinant erythropoietin (EPO) N-glycosylation, human milk oligosaccharides (HMOs), mucin-type O-glycans, gangliosides, and site-specific compositional data.

More recently, Glycowork [26] has been released as an open-source Python package for glycan-related data analysis and machine learning algorithms. It provides ~ 50,000 glycan sequences with ~ 35,000 species-related, ~ 14,000 tissues-related, and ~ 1,000 disease-related annotations. It also provides over 550,000 glycan-protein binding data. Those data are used in the deep learning model. NSequonPred, which is one of the trained models in Glycowork, helps users to predict whether the given N-sequon is glycosylated. The latest version of the Glycowork framework has enriched the motif annotation and expanded the model for the multiple glycomics expression data sets [27].

Table 1 summarizes the glycan biosynthetic tools described here. The “Applications” column in this table indicates the biological applications that have been illustrated iusing these tools. Many of these have studied human milk oligosaccharides (HMO), their biosynthetic pathways, and relevant enzymes. Glycologue in particular has recently published work on HMOs to predict important enzymes in their biosynthesis [11] as well as on a study of glycoside hydrolases to conversely study the potential pathogens associated in human gut [28]. Others have shown that their tools are able to predict glycosylation pathways given glycomics profiles, usually from mass spectrometry experiments; these have been indicated as “MS.”

**Table 1:** A summary of the selected software and tools described for glycan biosynthetic pathway predictions

Name	Glycan type	Function	Application type
Glycologue	*N*-, *O*-, HMO, gangliosides	Pathway prediction	Web tool
GlycoVis	*N*-	Glycan distribution and pathway prediction	Windows-based software tool (available by request)
Glycan Pathway Predictor (GPP)	*N*-	Pathway prediction	Web tool
UniCorn	*N*-	Glycosylation pathway database	*Currently closed due to inaccessibility of UniCarbKB*
GNAT	*N*-, *O*-	Pathway prediction	MATLAB (MathWorks)
GlyCompare	*N*-, *O*-, HMO, glycolipids	Pathway prediction and comparison	Python library
GlycoMME	*N*-	Pathway prediction based on glycomics data	MATLAB (MathWorks)
GlycoWork “network” module	Any linkage defined glycan data	Pathway prediction	Python library

Most tools have focused on the more well-studied glycan types, but recently, there has also been a report on a theoretical model for glycosaminoglycan biosynthesis [29]. While this has not been implemented in any tool, it can potentially be incorporated into any of the pathway modeling tools incorporating kinetic parameters. Machine learning has also been used for predicting protein glycosylation [30] but has yet been implemented as a tool for practical use.

### In silico simulations of glycosylation

In the field of glycoengineering, several attempts have been made to simulate glycosylation. As mentioned earlier, mathematical models have been proposed and further used to predict glycomes, comparing gene expression profiles and mass spectrometry glycomics datasets for validation [31, 32]. However, these models were based on Michaelis–Menten kinetics to model the reaction equations; many of the parameters for these reaction equations are often unknown, creating a bottleneck in simulating these models accurately. Various models have been proposed to emulate the Golgi apparatus, where the majority of glycogenes reside [30, 33]. Parameter estimation methods and sensitivity analysis tools have been applied to fill in these gaps, but validation has always been an issue.

**Fig. 3:**
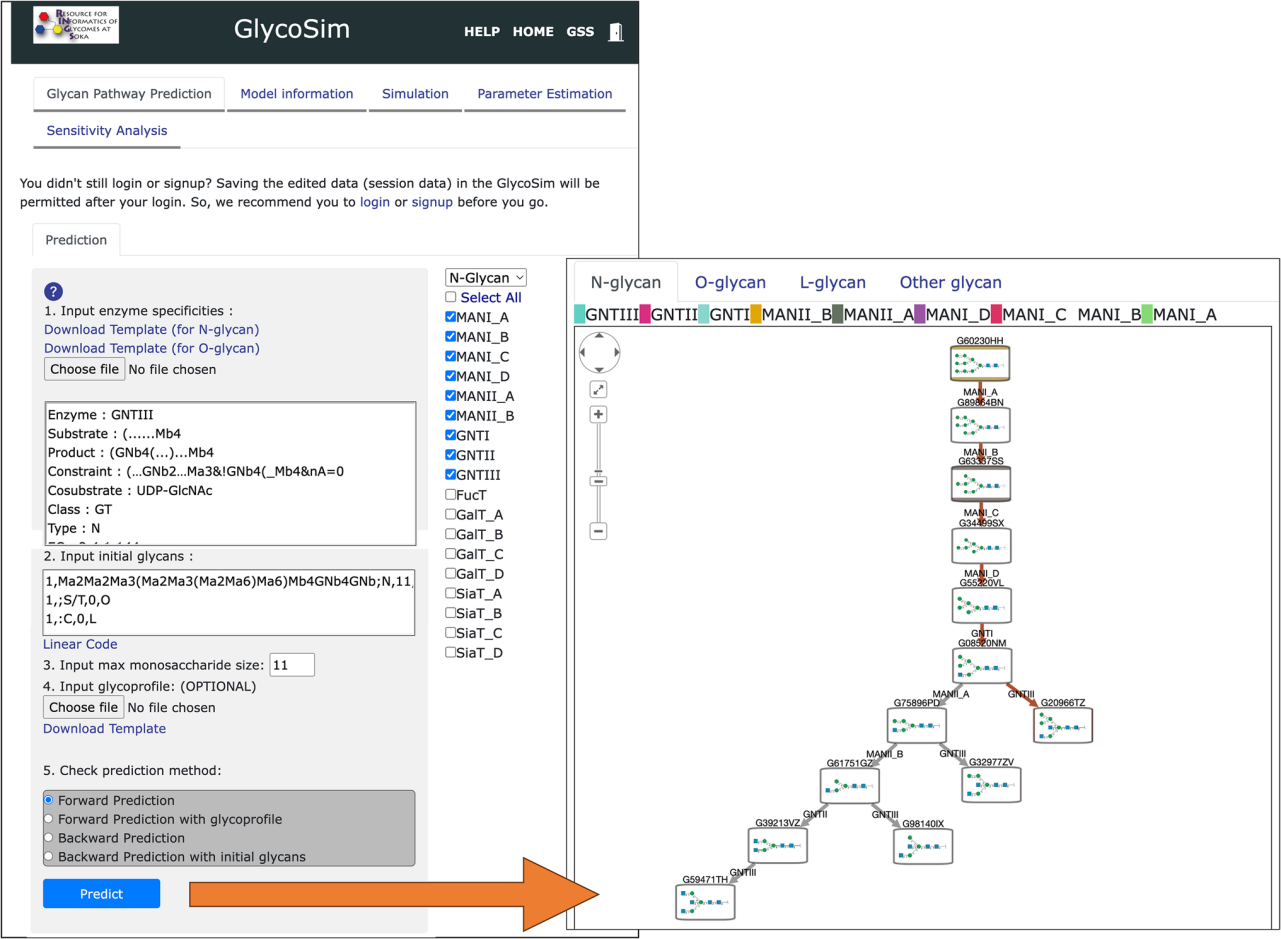
A snapshot of the results of performing biosynthetic pathway prediction in GlycoSim. Enzyme specificities can be modified to add or remove constraints to each enzyme. The predicted pathway is then stored as a mathematical model such that simulations can be performed on them

Nevertheless, we have been developing the GlycoSim tool (https://glycosim.rings.glycoinfo.org) [34] to provide a means for non-computational scientists to access these models and use them with their own data. GlycoSim uses the same functionality as the GPP tool to predict the glycosylation pathway given a substrate(s) and list of predefined glycogenes, but it also provides more flexibility in specifying substrate by using user-defined enzyme specificities. Figure 3 is a screenshot of the GlycoSim pathway prediction module, where step 1 for inputting enzyme specificities can be manually edited based on the LiCoRR rules [35]. Based on the predicted pathway (shown in the bottom of Fig. 3), a mathematical model is generated, where further parameters can be specified. We are also developing a database of predicted parameters for the reaction equations in these models. GlycoSim also has modules for parameter estimation of the missing parameters and sensitivity analysis of the parameters to determine the more sensitive parameters in the model. Such information can aid in determining the most important parameters in the model.

A large variety of parameter estimation methods are available in many software libraries, and optimization functionality is often used on top of the estimated parameters. In our work, we have previously attempted parameter estimation using the Particle Swarm and Simulated Annealing methods of COPASI [36] on glycomics and gene expression data from three types of mouse stem cells [37]. First, by using a normalized dataset of gene expression data for mouse ES cells, the *N*- and *O*-glycan biosynthesis pathways were predicted, and a mathematical model was generated using Systems Biology Markup Language (SBML) [38]. Then the model was imported into COPASI to test several parameter estimation methods. The estimated ranges were set initially to a very large range and, after repeated estimations, narrowed down to ranges to include the resulting values that were often estimated. This process was repeated 3–4 times. As a result, while the estimation process took an order of magnitude longer, Simulated Annealing was found to consistently produce stable values compared to Particle Swarm. The residual sum of squares (RSS) was used to estimate how well the model could reproduce the experimental results, and we found that when the RSS value was less than 1.0 × 10^−10^, the simulation results were quite close to the experimental data. The ES model was then tested on the other stem cell data from mouse, namely ExE and EB cells, using the estimated parameters from ES cells, where we could also obtain RSS values within 1.0 × 10^−9^ for *O*-glycans and 1.0 × 10^−6^ for *N*-glycans, indicating that we were successfully able to estimate these parameters well for *O*-glycans, but it was not as close for *N-*glycans. We found that in the glycomics data, there was a structure whose abundance could not be identified, thus resulting in a lower RSS value for the latter. However, conversely, we can claim that using RSS appears to be an effective method for scoring the fitness of a parameter set. In order to make this data available to the public, we are currently developing a database of these estimated parameters for others to test as well.

However, to avoid this missing parameter issue altogether, Boolean networks, Bayesian inference, Markov chain modeling, and other statistical methods have also been employed by others to mathematically calculate the parameters to reproduce glycan distributions without requiring kinetic information [25, 39, 40]. Flux analysis and multivariate data analysis are methods that attempt to capture bioprocesses more mechanistically, compared to the enzymatic methods which require many parameters [41, 42]. Moreover, a modeling framework based on genome reconstruction but using reaction flux flow stoichiometry, discretized variable state parameters, and mass balances has been developed, called DReaM-zyP, for discretized reaction network modeling using fuzzy parameters. This framework has been packaged into a tool called Glyco-Mapper which includes all CHO *N-*glycosylation genes, nucleotide sugar synthesis, transporter, and glycosylation-relevant metabolism genes [43]. It was shown to be able to model and predict many of the well-known CHO-engineered glycoforms published in the literature.

## Future outlook

In this brief review, we have introduced databases and software tools to enable the in silico prediction of glycosylation, mainly for mammalian cells. Many databases in the glycosciences have incorporated glycogene information to enable researchers better accessibility to such information. However, a centralized resource for such parameters is still a major need for the community. While more generalized databases for such parameters, such as BRENDA, exist, the substrate specificities are hard to define. The LiCoRR reaction rules based on LinearCode format, and the corresponding LiCoRRice format for IUPAC format, are considered the standards for such substrate specificities. We expect that databases providing such specificity information will be crucial to advance research in in silico glycosylation analysis.

Another issue is the lack of information on the localization of glycogenes especially at the compartmental level within the Golgi apparatus. Various models have been proposed in an attempt to simulate the Golgi, but research into the structure of the Golgi itself is still underway. Thus, the majority of the models presented in Table 1 have not considered multi-compartment models and are simply predictions of pathways disregarding localization. If localization is taken into consideration, it is currently only possible to estimate where specific enzymes reside within the Golgi apparatus. Therefore, while there are multi-compartment models developed and shown to be relevant for bioprocessing specific glycosylation patterns [33, 44], they are not available freely for expanded use by the community. Further research in click-chemistry [45] and cryo-EM [46] are making headway to identify enzyme localization at the subcellular level, but the transfer of any new insights into the informatics side still requires much effort. Resources to store bioimaging data and 3D structures of glycoconjugates exist, but they are often not annotated sufficiently to identify the glycan-related components involved.

Moreover, this Trends has mainly focused on mammalian systems, but much research has also progressed in understanding glycosylation in bacteria [47] and plants [48] as well. However, the database integration and in silico tools for these systems are yet to be fully developed. Many of the tools and databases described here have started to accumulate such data, but a user-friendly interface and infrastructure to enable plant and microbiologists to access and supplement these databases still needs to be constructed. Such developments would enable a better understanding of the roles of glycans in microbiomes and the environmental sciences. With the advancement of more high-throughput technologies and corresponding submission of high-quality data into databases and repositories, data-driven models can become more effective, especially with the remarkable development of large language models and AI technology.

In summary, there is much work to do in terms of bioinformatics, systems biology, microbiology, genome informatics, plant biology, etc. to better integrate the data produced and to develop user-friendly tools that allow researchers to access and analyze their data from a bird’s eye view. The GlySpace Alliance [49], Glycoinformatics Consortium (https://glic.glycoinfo.org), and Systems Glycobiology Consortium (https://sysglyco.org) are efforts to enable interactions between these heterogeneous fields aimed towards the same goal. The GlySpace Alliance consists of major glycan-based Web portals in the USA, Japan and Europe, where glycans, glycoproteins, and related metadata are shared freely. This alliance forms a basic informatics infrastructure for the glycosciences. The Glycoinformatics Consortium, or GLIC, is a group of glycoinformaticians who have developed databases and software for the glycosciences. Webinars and hackathons are held to enable interaction between glycoscience researchers and bioinformaticians, in an attempt to create synergy and more efficiently produce useful tools and data resources. Finally, the Systems Glycobiology Consortium, or SysGlyco, is a group of glycobiologists and informaticians interested in developing systems biology tools for the glycosciences. Many of the products of this consortium were presented in this review. These groups are currently unfunded and number few, but time will tell when the fruits of their labor will contribute to the glycosciences and the life and environmental sciences as a whole.
